# Sex-Specific Coronary Artery Calcium Score Thresholds Predictive of Obstructive Coronary Artery Disease: Ready for Prime Time?

**DOI:** 10.1016/j.jscai.2025.104113

**Published:** 2025-12-18

**Authors:** Khansa Ahmad, Islam Y. Elgendy

**Affiliations:** a Division of Cardiology, Boston Medical Center, Brighton, Massachusetts; b Division of Cardiovascular Medicine, Gill Heart and Vascular Institute, University of Kentucky, Lexington, Kentucky

**Keywords:** coronary artery calcium, sex differences

Coronary artery calcium (CAC) is a direct measure of subclinical calcified atherosclerotic plaque, and an established risk-enhancing factor for atherosclerotic cardiovascular disease risk stratification beyond traditional risk factors.^1^ It has been estimated that ∼17.6 million Americans may be eligible for CAC screening.^2^ CAC score, which was first described in 1990 by Agatson et al,^3^ represents a quantification of calcium in the coronary arteries by evaluating the number, areas, and peak density of calcified lesions on computed tomography (CT). CAC score is routinely reported for other noninvasive stress modalities such as single-photon emission CT and positron emission tomography.

The 2019 American College of Cardiology and American Heart Association guidelines for primary prevention of atherosclerotic cardiovascular disease recommend the integration of the CAC score in treatment decision-making among intermediate-risk individuals.^1^ Specifically, they recommend statin initiation if the CAC score is >100 irrespective of age, and 1 to 99 among those >55 years^1^; however, these guidelines do not provide sex-specific cutoffs for statin initiation. Indeed, studies have established that men are likely to have a nonzero calcium score by the age of 45 years,^4^^,^^5^ but women tend to have a higher risk of coronary events than men within the same CAC score categories.^4^

To date, studies have primarily focused on the role of the CAC score in guiding preventive therapy. Unlike aortic valve disease, in which a calcium score of ≥1300 in women and ≥2000 in men correlates with severe aortic stenosis,^6^ there is no defined CAC score cutoff value which predicts obstructive coronary artery disease (CAD). This is due, in part, to the shielding effect of calcium or the “blooming” artifact that prevents adequate intraluminal assessment on the coronary CT angiogram.

In this issue of *JSCAI,* Ozdemir et al^7^ examine the sex-specific CAC score thresholds that are predictive of obstructive CAD among 1799 consecutive patients who underwent invasive coronary angiography over a 5-year period using data from their large health system. The investigators demonstrated that a CAC score threshold of ≥1400 for men and ≥1000 for women was predictive of obstructive CAD at 90% specificity. There was no interaction based on the age groups (<65 vs >65 years) and the sex-specific CAC score threshold.

The findings by Ozdemir et al serve as a reminder that the diagnostic yield of noninvasive imaging modalities might be different in women and men; however, a few issues deserve careful consideration. First, this is a retrospective study from a large tertiary referral center; hence, the findings might not be generalizable to the wide-ranging individuals who undergo CAC screening. For example, the median age was 66 years, which is much older than that of individuals who undergo CAC screening for risk stratification.^1^ Second, the study enrolled patients who underwent invasive coronary angiography, which implies that they were likely symptomatic with a higher likelihood of obstructive CAD. The timeframe between the CAC score and invasive coronary angiogram was not specified. This is pertinent since plaque morphology may change over time.^8^ Third, the sensitivity of the described thresholds was quite low (ie, 27% and 26% in women and men, respectively), thus limiting the applicability of these findings as a screening tool for the broader population. Additionally, the positive predictive value was modest, which reflects the limited number of patients who had CAC scores above the defined thresholds. Finally, these thresholds were not compared with other noninvasive modalities, which are more widely used in clinical practice to screen for obstructive CAD. Notwithstanding these issues, the investigators should be applauded for their work, which represents the first step to identifying sex-specific CAC score thresholds that are predictive of obstructive CAD.

With the widespread use of CAC as a screening tool either separately or as a part of other noninvasive stress testing or noncardiac chest CT scan, the findings by Ozdemir et al could serve as a foundation for future investigations and applications. Prior data indicate that asymptomatic individuals with a CAC score >1000 have major adverse cardiovascular event rates similar to patients with stable established atherosclerotic cardiovascular disease.^9^ The thresholds identified by Ozdemir et al (ie, CAC score >1000 in women and >1400 in men) could serve as markers to consider prophylactic antiplatelet therapy and intensification of lipid-lowering therapies to achieve a low-density lipoprotein level <70 m/dL, even among asymptomatic individuals. However, these thresholds alone should not prompt downstream invasive coronary angiogram and/or revascularization, and these decisions should be guided according to the patient’s symptoms and clinical presentation.^10^

In conclusion, the study by Ozdemir et al is one step in establishing sex-specific thresholds for CAC scores predicting obstructive CAD. Future prospective studies with a generalizable population are necessary to validate these thresholds and evaluate the impact of secondary preventive measures, including antiplatelet and intensification of lipid-lowering therapies, on future cardiac events.

## Declaration of competing interest

The authors declared no potential conflicts of interest with respect to the research, authorship, and/or publication of this article.
